# SELDI-TOF-MS Proteomic Profiling of Serum, Urine, and Amniotic Fluid in Neural Tube Defects

**DOI:** 10.1371/journal.pone.0103276

**Published:** 2014-07-23

**Authors:** Zhenjiang Liu, Zhengwei Yuan, Qun Zhao

**Affiliations:** Department of Pediatric Surgery, The Shengjing Hospital, China Medical University, Heping District, Shenyang City, Liaoning Province, People’s Republic of China; University of Queensland, Australia

## Abstract

Neural tube defects (NTDs) are common birth defects, whose specific biomarkers are needed. The purpose of this pilot study is to determine whether protein profiling in NTD-mothers differ from normal controls using SELDI-TOF-MS. ProteinChip Biomarker System was used to evaluate 82 maternal serum samples, 78 urine samples and 76 amniotic fluid samples. The validity of classification tree was then challenged with a blind test set including another 20 NTD-mothers and 18 controls in serum samples, and another 19 NTD-mothers and 17 controls in urine samples, and another 20 NTD-mothers and 17 controls in amniotic fluid samples. Eight proteins detected in serum samples were up-regulated and four proteins were down-regulated in the NTD group. Four proteins detected in urine samples were up-regulated and one protein was down-regulated in the NTD group. Six proteins detected in amniotic fluid samples were up-regulated and one protein was down-regulated in the NTD group. The classification tree for serum samples separated NTDs from healthy individuals, achieving a sensitivity of 91% and a specificity of 97% in the training set, and achieving a sensitivity of 90% and a specificity of 97% and a positive predictive value of 95% in the test set. The classification tree for urine samples separated NTDs from controls, achieving a sensitivity of 95% and a specificity of 94% in the training set, and achieving a sensitivity of 89% and a specificity of 82% and a positive predictive value of 85% in the test set. The classification tree for amniotic fluid samples separated NTDs from controls, achieving a sensitivity of 93% and a specificity of 89% in the training set, and achieving a sensitivity of 90% and a specificity of 88% and a positive predictive value of 90% in the test set. These suggest that SELDI-TOF-MS is an additional method for NTDs pregnancies detection.

## Introduction

The prevalence of neural tube defects (NTDs) is known to vary significantly based upon geography and ethnicity, with ranges from 0.5 to 6 in 1,000 newborns [1]. The mothers of an NTD-affected child are 10-fold more likely to give birth to a second child with an NTD, suggesting the involvement of both environmental and genetic factors in their etiology. There are multiple types of isolated NTDs including spina bifida and anencephaly [2]. Current prenatal screening efforts are based on two complementary methods, maternal serum alpha-fetoprotein (MSAFP) and ultrasound screening. It has been determined that in a fetus with an NTD, exposed membranes allow AFP to leak into the amniotic fluid and then into maternal serum, at a level of roughly in proportion to the size of the exposed area. However, the level of MSAFP is not a specific indicator of an NTD, since it is also increased in ventral wall defects (omphalocele or gastroschisis), abnormal glomerular diseases such as nephrotic syndrome, defective placental membranes (fetal hydrops), and fetal blood contamination due to a traumatic amniocentesis, as well as other pregnancy-related problems [1]. Although a matter of some controversy, when the spina bifida lesion is covered with healthy skin, MSAFP and amniotic fluid AFP (AFAFP) concentrations are generally found to be normal. Therefore, due to the low specificity of MSAFP or AFAFP levels, its use as a screening tool, has limited diagnostic value. Norem and coworkers found that among the 102 NTDs cases who had received MSAFP testing, 25 cases (25%) had negative maternal serum screening results, including 15 (38%) of the 40 spina bifida cases tested, 6 (67%) of the 9 encephalocele cases tested, and 4 (8%) of the 53 anencephaly cases [3]. At present, there does not appear to be a more specific marker of NTDs that has been identified in maternal serum [1]. Kooper et al. found that 27 out of 6,188 pregnancies (0.4%) without any increased NTD risk had AFAFP levels >2.5 MoM (multiples of the median), two of which were associated with NTDs; two out of 258 pregnancies (0.8%) with an increased NTD risk had elevated AFAFP levels and were associated with affected pregnancies; and 44 of 55 pregnancies (80%) with clinically diagnosed fetal NTDs had an increased AFAFP levels [2].

Biochemical diagnosis of NTDs is based on the electrophoresis of amniotic fluid cholinesterases [4]. Cholinesterases includes butyrylcholinesterase, normally present in the serum and amniotic fluid, and acetylcholinesterase, which is specific to neural tissue but is normally absent from amniotic fluid. When the fetus has an NTD, acetylcholinesterase is present as a rapidly-migrating eletrophoretic band, in addition to butyrylcholinesterase. However, amniocentesis is an invasive procedure, and it has not been used routinely in the clinical practice.

Surface-enhanced laser desorption/ionization time-of-flight mass spectrometry (SELDI-TOF-MS) is a breakthrough in clinical proteomics, and can detect different protein expression patterns of body fluid and tissue specimens between patients and healthy subjects, and its rapid development provides an alternative tool to search for biomarkers. SELDI-TOF-MS detects proteins selectively adsorbed onto the surface of a protein chip array after non-specifically bound proteins are washed off by stringent buffers, and has been shown to be sensitive, rapid and reliable. This technique has been successfully applied in the discovery of serum biomarkers for many cancers [5]–[18], nervous system diseases [19]–[22] and renal diseases [23]–[32]. Furthermore, construction of a classification tree using “artificial intelligence” algorithms to process SELDI data improves the accuracy to differentiate cancer patients from non-cancer groups [33]–[34].

Our hypothesis is that protein expression profiles of NTDs may be considered as a potential diagnostic approach using SELDI-TOF-MS. The purpose of this pilot study was to preliminarily explore the differential protein expression pattern between NTD case mothers and normal control mothers using SELDI-TOF-MS protein profiling and Classification and Regression Tree (CART) analysis, in order to differentiate pregnancies complicated by the presence of an NTD-affected fetus from healthy controls.

## Methods

### Subjects and experimental design

Experimental protocol was approved by China Medical University Institutional Review Board. Written informed consent was obtained from all mothers with NTD-affected fetuses and healthy volunteers for this study. 120 maternal serum samples (64 mothers with NTD-affected fetuses and 56 normal control mothers), 114 maternal urine samples (61 mothers with NTD-affected fetuses and 53 normal control mothers) and 113 maternal amniotic fluid samples (61 mothers with NTD-affected fetuses and 52 normal control mothers) were collected between gestational weeks 15.0 and 34.0. Gestational age was determined from the date of the last menstrual period (LMP) or by an ultrasonography examination when the LMP was uncertain. Healthy volunteers receiving prenatal medical examination in The Shengjing Hospital were recruited into the control group. All of the normal control mothers, which had no pregnancy-related problems, such as diabetes or hypertension, had or were carrying fetuses lacking any congenital defects. All of the normal control mothers had normal biochemistry detection of serum and urine. Control amniotic fluid samples were collected from pregnant women with a normal fetal karyotype. Karyotypes of all fetuses were evaluated following amniocentesis. Prenatal diagnoses of NTD-affected and normal fetuses were confirmed using ultrasonography. NTDs in our study were classified into two different clinical types: spina bifida and anencephaly.

### Collections of Serum, Urine and Amniotic Fluid Samples

Two ml of whole blood was collected in the morning after fasting overnight and stored at 4°C for 1 hour. Blood samples were then centrifuged at 4,000 rpm, 4°C for 10 min and 100 µl aliquots were stored at −80°C until analyzed. Two ml of urine was collected in the morning after fasting overnight. Urine were immediately centrifuged at 2,500 rpm, 4°C for 5 min, and 100 µl aliquots were stored at −80°C until analyzed. Amniotic fluid were obtained from mothers carrying NTDs fetuses during transabdominal amniocentesis before induced labor, and mothers suspected of having a fetus with trisomy 21 or 18 that were ultimately confirmed as a normal karyotype during the prenatal examination. Amniotic fluid samples were collected during an aseptic procedure through the abdominal wall and by direct ultrasound guidance using a 21-gauge puncture needle. Two ml of amniotic fluid were collected, immediately centrifuged at 2,500 rpm, 4°C for 5 min, distributed into 100 µl aliquots and stored at −80°C until analyzed.

### ProteinChip CM10 Array

The efficacies of both strong anion exchange and weak cation exchange (WCX) protein chips (Ciphergen Biosystems, Inc., Fremont, CA, USA) for serum protein profiling were tested in this study. Results showed that the weak cationic exchange ProteinChip, CM10, had a higher capture ability and signal-to-noise ratio (S/N). Therefore, CM10 was selected for all subsequent experiments. Binding of proteins to the ProteinChip CM10 array can also be affected by pH dependent of the buffer and changing the ionic strength of the buffer.

### Sample Preparation

Serum, urine or amniotic fluid samples were thawed on ice and centrifuged at 10,000 rpm for 2 min at 4°C, at which time 20 µl U9 buffer (9 mol/L urea, 2% 3-[(3-cholamidopropyl) dimethylammonio] propanesulfonate, 50 mmol/L Tris–HCl, pH 9.0, and dithiothreitol) was added into 10 µl supernatant, and vortexed for 30 min at 4°C. At this point, 360 µl binding buffer (50 mmol/L NaAC, pH 4.0) was added into serum sample, 250 µl of binding buffer was added into urine sample, 170 µl of binding buffer was added into amniotic fluid sample, and immediately mixed. To equilibrate the chip, 200 µl binding buffer was applied to each chip spot, shaking at 250 rpm for 5 min and excess buffer was removed without contacting the active surface. The same procedure was repeated once. 100 µl prepared serum sample, or urine sample, or amniotic fluid sample, was then added into each Bioprocessor well, and chip was incubated in a humidity chamber for 1 h, shaking at 250 rpm at room temperature. Excess buffer was removed. 200 µl binding buffer was added into each well, shaking at room temperature for 5 min. The above procedure was repeated once. Each spot was washed with 200 µl deionized water. Then 0.5 µl saturated sinnapinic acid was applied to each spot and the chips were allowed to air dry. Chips were re-incubated with sinnapinic acid and air dried again. Captured proteins by the arrays were detected on a PBS-II C reader (Ciphergen Biosystems).

### Mass spectrum detection

The accuracy of mass spectrum was calibrated using the all-in-one peptide molecular mass standard (Ciphergen Biosystems, Inc., Fremont, CA, USA) at the day of experiment. The high cutoff molecular weight was set as 50,000 daltons (Da), with an optimized range of 1,000 to 30,000 Da, laser intensity of 225 and detection sensitivity of 9. Each sample was activated 130 times. Error range of molecular weight was controlled at <0.1%. Intrachip and interchip quality controls were included. The coefficient of variation (CV) was controlled at <10% for peak amplitude.

### Screening of Differentially Expressed Protein by Software

All spectra were analyzed using Biomarker Wizard Software (Ciphergen Biosystems, Inc.). Qualified mass peaks (S/N >5) with *m/z* between 1000 and 30000 Da were detected automatically and majority of resolved protein/peptides were within this range. Molecular masses from 0–1000 Da were excluded from analysis since they were mainly noises from the energy-absorbing molecule (EAM). Peak intensities were normalized to the total ionic current of peaks with *m/z* between 1000 and 30000 Da using ProteinChip software version 3.1.1 (Ciphergen Biosystems, Inc.). In a validation experiment, protein peaks should present in more than 10% samples and deviation of peak amplitude should be smaller than 0.3% in each sample. Parameters of protein peaks were presented as mean value plus or minus standard deviation and spectral data of the training set were further analyzed using Biomarker Pattern Software (BPS) (version 5.0; Ciphergen Biosystems) to establish a classification tree.

### Classification and Regression Tree (CART) Analysis

Decision tree classification pattern was generated using BPS version 5.0, a statistical tool developed by Breiman et al. [35] to implement CART. BPS uses peak information generated from the sample training set to build the classification tree. CART consisted of tree construction and tree pruning [36], [37]. The tree construction process primarily splits spectral data of the training set into two nodes by questionnaire criteria. The splitting decision is based on the intensity of a peak. The answer to “Does mass A have intensity less than or equal to X” splits the data set into two nodes, a left node for yes and a right node for no. The splitting process was repeated until no further gain in classification and terminal nodes were reached. Further classification of terminal nodes is based on the clinic grouping of most samples (i.e., NTD and control) that represents the majority of samples in that node. In the tree pruning step, the classification tree is cut down to a desired size using tree-cost complexity pruning [33].

### Statistical Analysis

Calculation of sensitivity, specificity, positive predictive value and negative predictive value were performed using BPS version 5.0 (Ciphergen Biosystems, Inc.). Comparison of parameters between NTD group and control group were performed using the *t*-test. *P*<0.05 was considered statistically significant.

## Results

### Serum SELDI Profiles of NTDs versus Normal Controls

**Figure 1:**
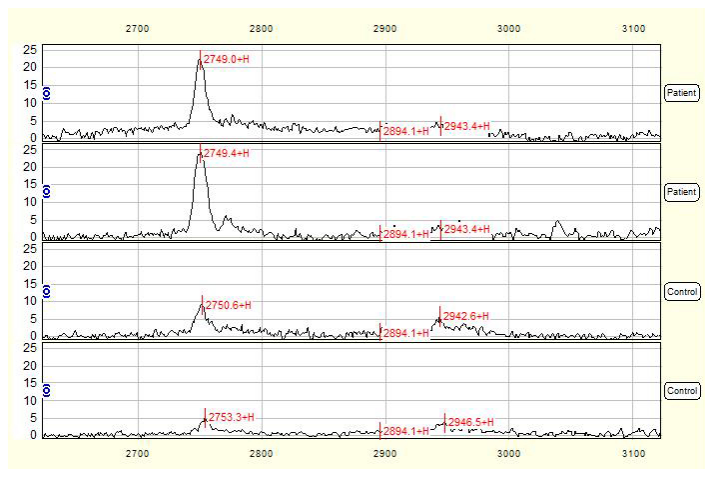
SELDI profile showed a peak with average mass of 2750-regulated in NTDs compared with control serum. Serum mass spectra of 2 mothers with NTD-affected fetuses were compared with those of 2 control mothers and the mass-to-change ratio (m/z) ranged between 2700–3100 Da. X-axis showed molecular weight of peaks and Y-axis showed intensity of peaks.

**Figure 2:**
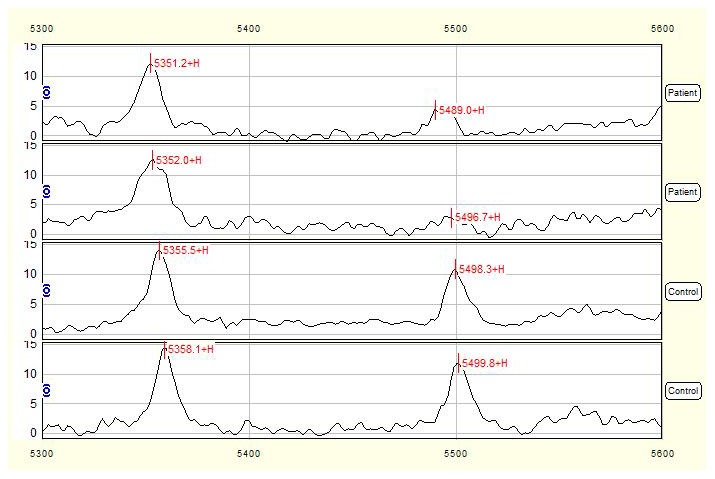
SELDI profile showed a peak with average mass of 5497-regulated in NTDs compared with control serum. Serum mass spectra of 2 mothers with NTD-affected fetuses were compared with those of 2 control mothers and the mass-to-change ratio (m/z) ranged between 5300–5600 Da. X-axis showed molecular weight of peaks and Y-axis showed intensity of peaks.

Total of 55 qualified mass peaks (S/N >5) were detected. SELDI was particularly effective in resolving low molecular weight (<10 kDa) proteins and polypeptides. The intensities of 12 of 55 mass peaks between NTDs group and control group were significantly different, among which 8 proteins with *m/z* of 4105, 4297, 4188, 6650, 8583, 3282, 2750 (Fig. 1) and 3327 Da were up-regulated, while 4 peaks with *m/z* of 5497 (Fig. 2), 28078, 9155 and 9434Da were down-regulated in NTDs group compared to normal control group (Table 1).

**Table 1:** Proteomic Features Showing Significantly Differences in Expression by Serum ProteinChip in Detection of Mothers with NTD-Affected Fetuses and Control Mothers.

Mass(Da)	P value	NTDs (Mean ±SD)	Ctrl (Mean ±SD)	Fold
4105	0.000163	59.34±8.53	47.18±5.73	1.26
4297	0.000641	35.82±3.62	22.83±3.60	1.57
4188	0.00196	40.20±5.08	25.56±4.52	1.57
5497	0.00376	4.19±0.20	7.97±0.73	0.53
28078	0.00483	5.41±0.92	6.61±0.89	0.82
6650	0.00616	66.75±5.72	55.37±4.20	1.21
8583	0.00695	47.28±3.31	39.47±2.63	1.20
3282	0.0155	8.64±1.06	5.03±1.01	1.72
2750	0.0172	18.28±1.30	12.19±1.76	1.50
9155	0.0237	10.16±2.35	13.51±2.30	0.75
3327	0.0390	7.24±0.78	5.65±0.08	1.28
9434	0.0390	11.91±1.84	15.97±1.49	0.75

NTDs: Neural Tube Defects.

Mean± SD refers to the peak intensities.

### Urine SELDI Profiles of NTDs versus Normal Controls

**Figure 3:**
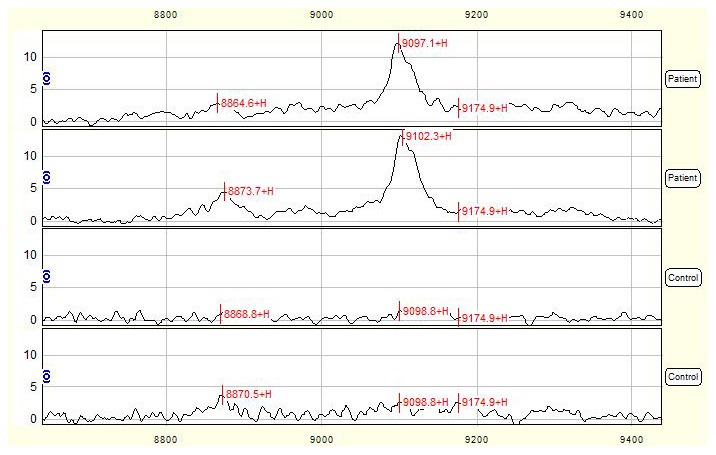
SELDI profile showed a peak with average mass of 9099-regulated in NTDs compared with control urine. Urine mass spectra of 2 mothers with NTD-affected fetuses were compared with those of 2 control mothers and the mass-to-change ratio (m/z) ranged between 8800–9400 Da. X-axis showed molecular weight of peaks and Y-axis showed intensity of peaks.

Total of 39 qualified mass peaks (S/N >5) were detected. The intensities of 5 of 39 mass peaks were significantly different between NTDs group and normal control group, among which 4 proteins with *m/z* of 8320, 8209, 9099 (Fig. 3) and 10567 Da were up-regulated, while 1 peak with *m/z* of 3458 Da was down-regulated in NTDs group compared to control group (Table 2).

**Table 2:** Proteomic Features Showing Significantly Differences in Expression by Urine ProteinChip in Detection of Mothers with NTD-Affected Fetuses and Control Mothers.

Mass(Da)	P value	NTD (Mean ±SD)	Ctrl (Mean ±SD)	Fold
8320	0.0167	5.72±0.55	3.50±0.77	1.63
8209	0.0198	10.34±1.12	6.89±0.98	1.50
3458	0.0275	1.62±0.05	12.00±0.95	0.13
9099	0.0376	8.80±0.76	5.02±0.27	1.75
10567	0.0376	1.63±0.03	0.83±0.01	1.96

NTD: Neural Tube Defect.

Mean± SD refers to the peak intensities.

### Amniotic Fluid SELDI Profiles of NTDs versus Normal Controls

**Figure 4:**
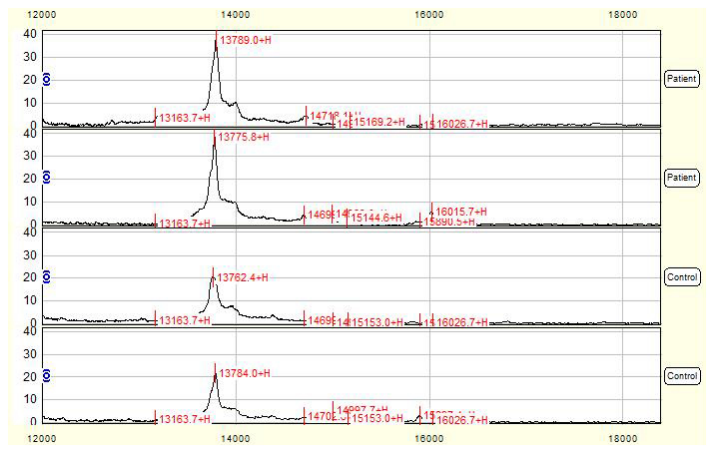
SELDI profile showed a peak with average mass of 13776-regulated in NTDs compared with control amniotic fluid. Amniotic fluid mass spectra of 2 mothers with NTD-affected fetuses were compared with those of 2 control mothers and the mass-to-change ratio (m/z) ranged between 12000–18000 Da. X-axis showed molecular weight of peaks and Y-axis showed intensity of peaks.

Total of 35 qualified mass peaks (S/N >5) were detected. 7 of 35 mass peaks in NTDs group have significantly different intensity to that of normal control group, among which 6 proteins with *m/z* of 14700, 7995, 15891, 16027, 13776 (Fig. 4) and 11040 Da were up-regulated, while 1 peak with *m/z* of 23417 Da was down-regulated in NTDs group compared to control group (Table 3).

**Table 3:** Proteomic Features Showing Significantly Differences in Expression by Amniotic Fluid ProteinChip in Detection of Mothers with NTD-Affected Fetuses and Control Mothers.

Mass(Da)	P value	NTD (Mean ±SD)	Ctrl (Mean ±SD)	Fold
14700	0.00601	3.13±0.02	1.42±0.01	2.20
7995	0.0181	3.62±0.21	1.67±0.13	2.16
15891	0.0181	4.88±0.06	0.54±0.01	9.02
16027	0.0181	3.86±0.07	0.38±0.01	10.27
23417	0.0253	5.90±0.92	7.28±1.49	0.81
13776	0.0350	31.97±2.26	25.17±1.90	1.27
11040	0.0476	5.77±0.05	1.33±0.01	4.34

NTD: Neural Tube Defect.

Mean± SD refers to the peak intensities.

### CART Analysis of Serum, Urine and Amniotic Fluid SELDI Profile

No single peak was identified alone, indicating that no peak could completely differentiate the NTD group from the normal control group for serum, urine and amniotic fluid samples, respectively.

### CART Analysis of Serum SELDI Profile

**Figure 5:**
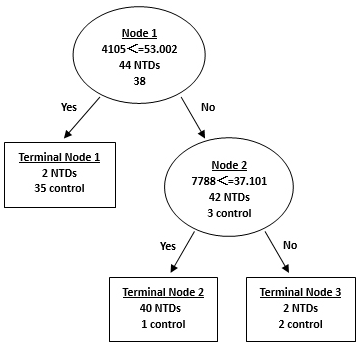
Diagram of a decision tree for the classification of serum samples from mothers with NTD-affected fetuses and control mothers. Circles indicated primary nodes and squares indicated terminal nodes. The mass value in the root nodes was followed by ≤ the intensity value.

A decision tree classification algorithm was built using all 55 protein peaks and 2 protein peaks at 4105 and 7788 Da were automatically selected as splitters. The 4105 Da peak was used as the root node in the classification tree to split all samples into two groups (Fig. 5): the left node (terminal node 1) included peaks with intensity ≤53.002 and the right node (node 2) contained the remaining peaks with intensity >53.002. Each branch node was then further classified into next layer using the same method with 7788 Da as the cutoff. Finally, all samples were classified into 3 terminal nodes and a classification tree was obtained (Fig. 5). This pattern analysis process yielded a sensitivity of 91%, specificity of 97%, positive predictive value of 98% and negative predictive value of 90% in the training set (Table 4).

**Table 4:** Performance of the Classification Tree Analysis of NTDs in Training and Test Sets of Serum Samples.

	Sensitivity,%	Specificity,%	Accuracy,%	PPV	NPV
Training set	91 (40/44)	97 (37/38)	94 (77/82)	98 (40/41)	90 (37/41)
Test set	90 (18/20)	94 (17/18)	92 (35/38)	95 (18/19)	89 (17/19)

NTDs indicates neural tube defects.

PPV indicates positive predictive value.

NPV indicates negative predictive value.

The validity and accuracy of the classification tree algorithm were then evaluated by challenging to classify blinded objects correctly in the test set, which consisted of 20 samples of serum from mothers with NTD-affected fetuses, 18 samples of serum from healthy controls. The algorithm correctly classified 92% (35 of 38) of the testing samples with a sensitivity of 90% (18 of 20), specificity of 94% (17 of 18), positive predictive value of 95% (18 of 19) and negative predictive value of 89% (17 of 19) (Table. 4).

### CART Analysis of Urine SELDI Profile

**Figure 6:**
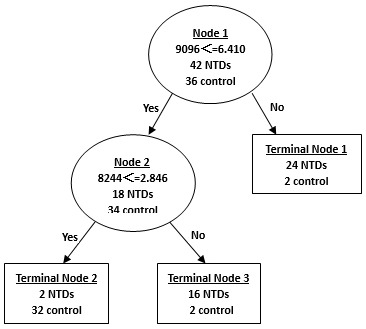
Diagram of a decision tree for the classification of urine samples from the mothers with NTD-affected fetuses and control mothers. Circles indicated primary nodes and squares indicated terminal nodes. The mass value in the root nodes was followed by≤ the intensity value.

Analysis of urine samples from mothers of NTD-affected fetuses and normal control mothers revealed two major, differentially expressed proteins at 9096 and 8244 Da used in the classification pattern to generate 3 terminal nodes (Fig. 6). Our results yielded sensitivity of 95%, specificity of 89%, positive predictive value of 91% and negative predictive value of 94% (Table 5).

**Table 5:** Performance of the Classification Tree Analysis of NTDs in Training and Test Sets of Urine Samples.

	Sensitivity,%	Specificity,%	Accuracy,%	PPV	NPV
Training set	95 (40/42)	89 (32/36)	92 (72/78)	91 (40/44)	94 (32/34)
Test set	89 (17/19)	82 (14/17)	86 (31/36)	85 (17/20)	88 (14/16)

NTDs indicates neural tube defects.

PPV indicates positive predictive value.

NPV indicates negative predictive value.

The validity of the classification tree was then challenged with a blind test set including another 19 samples of urine from mothers with NTD-affected fetuses and 17 samples of urine from healthy controls. The algorithm correctly classified 86% (31 of 36) of the testing samples with a sensitivity of 89% (17 of 19), specificity of 82% (14 of 17), positive predictive value of 85% (17 of 20) and negative predictive value of 88% (14 of 16) (Table. 5).

### CART Analysis of Amniotic Fluid SELDI Profile

**Figure 7:**
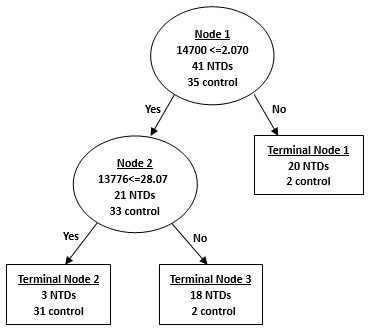
Diagram of a decision tree for the classification of amniotic fluid samples from the mothers with NTD-affected fetuses and control mothers. Circles indicated primary nodes and squares indicated terminal nodes. The mass value in the root nodes was followed by ≤ the intensity value.

Analysis of amniotic fluid samples from mothers of NTD-affected fetuses and normal control mothers revealed two major, differentially expressed proteins at 14700 and 13776 Da used in the classification pattern to generate 3 terminal nodes (Fig. 7). Our results yielded sensitivity of 93%, specificity of 89%, positive predictive value of 90% and negative predictive value of 91% (Table. 6).

The validity of the classification tree was then challenged with a blind test set including another 20 amniotic fluid samples from mothers with NTD-affected fetuses and 17 amniotic fluid samples from healthy controls. The algorithm correctly classified 89% (33 of 37) of the testing samples with a sensitivity of 90% (18 of 20), specificity of 88% (15 of 17), positive predictive value of 90% (18 of 20) and negative predictive value of 88% (15 of 17) (Table. 6).

**Table 6:** Performance of the Classification Tree Analysis of NTDs in Training and Test Sets of Amniotic Fluid Samples.

	Sensitivity,%	Specificity,%	Accuracy,%	PPV	NPV
Training set	93 (38/41)	89 (31/35)	91 (69/76)	90 (38/42)	91 (31/34)
Test set	90 (18/20)	88 (15/17)	89 (33/37)	90 (18/20)	88 (15/17)

NTDs indicates neural tube defects.

PPV indicates positive predictive value.

NPV indicates negative predictive value.

### Comparison of *m/z* of Serum, Urine and Amniotic Fluid SELDI Profile

Comparing among serum, urine and amniotic fluid SELDI profile, four differentially expressed masses with the same *m/z* of 4188, 6451, 11744 and 23425 Da, coexists in the serum, urine and amniotic fluid (Table 7).

**Table 7:** The same peaks of serum, urine and amniotic fluid proteomic profile of mothers with NTD-affected fetuses.

Serum (Da)	Urine (Da)	Amniotic fluid (Da)
4188	4191	4190
6451	6451	6453
11744	11760	11755
23425	23461	23417

## Discussion

MSAFP, AFAFP, and amniotic fluid cholinesterases are the primary protein biomarkers used in the prenatal diagnosis for NTDs [1]. MSAFP screening and ultrasonography are both used for prenatally detecting NTD-affected fetuses. Although The American College of Obstetricians and Gynecologists recommended that all pregnant women should be offered MSAFP screening in the second trimester of pregnancy, the detection rate for NTDs using elevated MSAFP level as a screening tool was only 75% to 80% [3]. Causes for elevated MSAFP levels other than NTDs include underestimated gestational age, congenital skin defects, pilonidal cysts, abdominal wall defects, gastrointestinal defects, obstruction, liver necrosis, cloacal exstrophy, cystic hygroma, sacrococcygeal teratoma, renal anomalies, urinary obstruction, polycystic kidney, absent kidney, congenital nephrosis, osteogenesis imperfecta, low birth weight, oligohydramnios, multiple gestation and maternal underweight [38]. Szajkowski et al. suggested that MSAFP screening had a low sensitivity for fetal hydrocephalus and was rarely elevated in isolated cases [39]. Screening for NTD is now a routine prenatal test, mainly due to its association with second-trimester maternal serum screening for Down syndrome determined by combination of low AFP value and high hCG value. AFAFP can also be used as an indicator for NTDs, but its specificity is not satisfactory [1], and sensitivity is 80% [2]. Therefore, the suggestion of performing routine AFAFP test in early second-trimester genetic amniocentesis by Widlund et al. [40] to rule out the possibility of an open fetal NTD, does not seem to be justified given its limited clinical diagnostic value. Electrophoresis of amniotic fluid cholinesterases can also be used to biochemical diagnose of NTDs, which has good sensitivity, specificity, positive predictive value and negative predictive value [4]. However, as an invasive technique, amniocentesis might cause abortion, infection, injury to mother and fetus, which must all be weighed against the value of performing the test.

Proteomic analysis provides a unique tool for the identification of diagnostic biomarkers, evaluation of disease progression and development of drugs [41]. SELDI-TOF-MS has been used to resolve proteins in biological specimens through binding to biochemically distinct ProteinChips [34], [42], and has many other advantages compared with traditional approaches: 1) it is much faster to perform; 2) it has high-throughput capability; 3) it requires only small amount of protein sample; 4) it has relatively high sensitivity to detect proteins at picomole to attamole range; 5) it can effectively resolve low mass proteins (2–20 KDa) and 6) it is directly applicable for development of clinical assays [26].

Since NTDs are multifactorial traits, it is likely that a combination of several biomarkers will become a signature which is necessary to effectively differentiate NTDs from controls, such as the combination of peaks at 4105 and 7788 Da in the serum samples, peaks at 9096 and 8244 Da in the urine samples, and peaks at 14700 and 13776 Da in the amniotic fluid samples. In our study, profiling of multiple proteins was performed using SELDI technology, and Biomarker Patterns Software was used to analyze the large volume of generated data. Proteins/peptides derived from both the fetus and the mother could be used in this classification system only if they produce a relatively accurate diagnosis, that is, these biomarkers can be detected by SELDI and accurately selected by the classifier.

Our results further confirmed the suitability of SELDI-TOF-MS for protein profiling of serum, urine and amniotic fluid samples, which yielded relatively high sensitivity and specificity for the diagnosis of NTDs. There were a greater number of different proteins in the serum samples than the amniotic fluid samples, when the amniotic fluid is more directly measuring fetal proteins than maternal serum. However, when we analyzed the data, we found that only four protein/peptide biomarkers, 4188, 6451, 11744 and 23425 Da, were detected in all serum, urine and amniotic fluid samples. One possible explanation is that these four protein/peptide biomarkers might be related to the abnormal protein expression of NTDs. Since identification of these biomarkers is essential for understanding their biological roles in NTDs, we are currently making efforts to characterize these protein/peptide biomarkers. The ultimate clinical application of protein profiling is for the early detection of NTDs from the maternal body fluid, such as serum or urine. Detection of larger number of peaks in serum samples than those of urine and amniotic fluid samples suggested that the identification of candidate biomarkers from maternal serum samples may be productive.

It was noted that not all differentially expressed protein peaks were used in the CART analysis. Wadsworth et al. suggested that CART analysis should examine all possible protein peak combinations in input spectral data to generate the best classification tree, in which any statistically insignificant protein peak included was also important for the classification algorithm after stratification [43]. Although peak of 7788 Da shown in Figure 5 had no statistical difference between the two groups of serum, it was crucial for the classification tree to delineate subsets of groups that had been stratified by the significant peak of 4105 Da. The use of peak of 8244 Da in the urine sample CART shown in Figure 6 was similar with that of peak of 7788 Da in the serum sample CART. According to literature, another research group has used two-dimensional gel electrophoresis (2-DE)/mass spectrometry (MS) to characterize differentially expressed proteins in amniotic-fluid samples (AFSs) of embryonic day (E) 17.5 rat fetuses with spina bifida aperta induced by retinoic acid (RA) [44]. They identified five proteins differentially expressed in AFSs of spina bifida aperta, including three upregulated proteins (transferrin, alpha-1 antiproteinase and signal recognition particle receptor, B subunit [SRPRB] 55 kDa), two downregulated proteins. Specifically, they found 11 alpha-1 fetoprotein (AFP) fragments that were downregulated and 35 AFP fragments that were upregulated in AFSs from embryos with spina bifida aperta. The comparative proteomic study of AFSs from rat fetuses with spina bifida aperta may provide new insights in neural tube defects and contribute to the prenatal screening.

This study suggests that NTDs-specific proteomic signatures are likely to present in serum, urine, and amniotic fluid of mothers carrying a NTDs fetus. It is reported that at present although AFP screening detects 88% of affected fetuses with a false positive rate of 3% in the second trimester, ultrasound has 100% specificity and 98% sensitivity for NTD at 18–20 week [45], [46]. Currently, it is still controversial whether or not it is sufficient to rely only on ultrasound to detect NTD. Recent literature has even suggested only ultrasound be used. New techniques in ultrasound have high specificity and sensitivity [47]. In the paper, there is a recommendation to abolish MSAP and rely only on ultrasound (which approaches 100% specificity and sensitivity). However, on the other hand, some recent studies also demonstrated the limitation of ultrasound approach alone to detect NTD. For example, one study showed that ultrasound biparietal diameter alone would detect 50% of cases for a 5% false-positive rate or 63% for 10%; adding AFP increases detection by 2%; and a combined test with Ultrasound biparietal diameter, AFP, and free b-hCG detects 58% for 5% or 70% for 10% [48]. Another study indicated that there were 20 perinatal deaths from NTD using ultrasound approach alone that could potentially have been prevented through the use of pre-conceptual folate [49]. Therefore, in New Zealand, new guidelines were implemented in 2010 that required all eligible pregnant women to be offered a nuchal translucency scan combined with a blood test to improve NTD detection. In our study, we could only take samples from those pregnant women who have already been diagnosed with NTD-affected pregnancies by prenatal ultrasound. If ultrasound is not sufficient enough for NTD in some cases, we think, SELDI-TOF-MS can at least serve as an additional method for NTDs pregnancy detection because of its relatively high sensitivity and specificity. Our pilot study demonstrated the application of SELDI protein profiling in the detection of mothers with NTD-affected fetuses. We have preliminarily established a relatively specific procedure to detect NTD-affected pregnancies using a combination of ProteinChip technology and Classification and Regression Tree Analysis.
